# Differential gene expression and identification of growth-related genes in the pituitary gland of South African goats

**DOI:** 10.3389/fgene.2022.811193

**Published:** 2022-08-22

**Authors:** Keabetswe T. Ncube, Edgar F. Dzomba, Ben D. Rosen, Stephen G. Schroeder, Curt P. Van Tassell, Farai. C. Muchadeyi

**Affiliations:** ^1^ Biotechnology Platform, Agricultural Research Council, Pretoria, South Africa; ^2^ Discipline of Genetics, School of Life Sciences, University of Kwa-Zulu Natal, Scottsville, South Africa; ^3^ Animal Genomics and Improvement Laboratory, Agricultural Research Service, United States Department of Agriculture, Beltsville, MD, United States

**Keywords:** transcriptome, differential gene expression, pituitary, goats, growth, carcass quality

## Abstract

Growth and carcass quality are economically important traits in goat production. This study investigated differentially expressed genes from the caprine pituitary gland transcriptome of South African indigenous goat breeds of varying growth performances and carcass quality parameters. Tissues were harvested from the pituitary gland of three South African Boer goats and three village ecotype goats all raised under similar conditions simulating intensive commercial production systems. Three additional tissues were harvested from village ecotype goats that were raised extensively on village farms. Between breed differences were investigated by comparing differential gene expression among three South African Boer and three village goats that were both raised under intensive commercial production system at a research farm. Within-breed differences were investigated by comparing differential gene expression among three village goats raised under extensive conditions (on-farm in Pella, S.A. village farming community) and three village goats raised under intensive commercial production system (at ARC research farm in Pretoria, South Africa. Total RNA was isolated from the pituitary gland of 36-week-old animals (*n = 9*) and sequenced individually in triplicates. An average of 28,298,512 trimmed, and quality-controlled reads/animal were mapped to the goat genome (*Capra_hircus.ARS1.94*) using HiSat2 software. Transcript assembly and quantification yielded 104 differentially expressed genes for village goats raised under extensive system and 62 for village goats raised under the intensive production system at the false discovery rate (FRD) of ≤0.05 and a fold change of ≥2. Growth-related genes such as *POU3F4* and *TSHZ1* were highly expressed within breeds raised under both production systems. Conversely, growth-related genes such as *FGFR2* and *SMPX* genes were highly expressed between breeds raised under similar production systems. Ballgown analysis revealed a high expression of *GH1* and *IGF1* in the intensively raised compared to extensively raised goats. Both genes were also highly expressed in the village goats when compared to the Boer. The differential gene expression data provided insights into genes and molecular mechanisms associated with growth and growth development in goats.

## Introduction

Growth and carcass quality are essential traits in goats and other domestic livestock species raised for the provision of meat. Variations in growth performance have been observed between and within goat breeds (Ncube, 2016). Goats in communal areas have been reported to have low growth performance which has been linked to the absence of selection programs in these regions (Masika and Mafu, 2004). Growth performance in South African goats is constrained, as majority (over 60%) of the goat farming is practiced in impoverished, marginalized communities (Dzomba et al., 2018) where farmers face various production challenges that affect growth and other traits of economic importance.

South Africa is represented by two goat production systems of commercial/intensive and extensive (Mdladla et al., 2017). The commercial/intensive production system is characterized by specialized breeds, well-designed housing systems, adequate feed, feed supplementation and regular health care systems (Mdladla et al., 2016). Hence breeds such as the South African Boer (SAB) raised in the commercial production system for meat production are reported to have high growth performance and meat yield (National Agricultural Marketing Council, 2005). Non-descript village goats, on the other hand, reared for multiple uses ranging from food security through provision of meat and socio-cultural roles are raised under the extensive production system characterized by low input management practices often associated with suboptimal nutrition and housing, inadequate veterinary services and lack of breed/animal improvement programs (Masika and Mafu, 2004; Gwaze et al., 2009). Village goats fend for food and sometimes travel long distances to browse in undesirable climatic conditions. The use of non-descript breeds coupled with the harsh conditions affects growth performance leading to low mature weights and lean animals. Village goat populations that are kept and adapted to most of these harsh production conditions are small with low growth rates when compared to the commercial populations (Masika and Mafu, 2004). However, high variability in growth rates and mature weights has been observed in village goat populations (Visser et al., 2004) indicating that some individuals may be better performers than typical animals in these communal farms, thus providing room for within-population selection. Unfortunately, there are limited records or information on production performance of goats from most communal farming systems (Masika and Mafu, 2004). With limited information, it is challenging to develop breeding programs that enable selection for growth rates and other traits of economic importance.

For optimal breed management and utilization, it is crucial to investigate and understand the genes and genetic pathways that play a role in growth and growth-related traits of goats and other domestic animals. The pituitary gland dubbed the “master gland”, secretes hormones that control other parts of the endocrine system and is the most studied in livestock because of its importance in processes such as growth, reproduction, stress, and lactation (Ooi et al., 2004). The pituitary is responsible for controlling growth at different developmental stages that involve interactions of numerous hormones and growth factors playing a role in endocrine and paracrine functions (Pareek et al., 2016). Therefore, investigating gene expression in the pituitary gland should aid in unravelling the genetic mechanisms influencing growth and carcass quality in South African indigenous goats raised under different production systems. Genes such as the growth hormone (*GH*), growth hormone receptor (*GHR*), insulin like growth factor I (*IGF-I*), leptin (*LEP*), caprine pituitary specific transcription factor-1 (*POU1F1*), caprine myostatin (*MSTN*), bone morphogenetic protein (*BMP*), and others may play important roles in growth (Alakilli et al., 2012). For example, mutations in *POU1F1* were associated with dwarfism in mice and humans, while polymorphisms in this gene are associated with production traits in goats (Lan et al., 2007). Some of the genes like MSTN are responsible for “double muscling,” a phenotype with significant muscle hypertrophy in sheep and cattle (Gan et al., 2008). Knowledge of such genes and their expression in different populations will shed light on growth- and growth-related traits that will in turn empower actions on breed improvement.

RNA sequencing is a high-throughput sequencing method for characterizing gene expression in specific tissues. This technology provides accurate counts of transcripts to measure relative expression and to discover new exons or genes (Wang et al., 2009). This approach is widely used for mapping, and quantifying transcriptomes and analyze gene expressions in various tissues (Cánovas et al., 2010). Lin et al. (2017) identified using RNA sequencing, genes differentially expressed at different postnatal growth stages in goats. Through transcriptome analysis, studies have been able to associate expressed genes to pathways that play a role in cell stimulations and neutral growth as well as phenotypes in slow and fast-growing chickens (Wu et al., 2018). These among other studies show potential for RNA-Seq as a tool to be used for gene expression profiling. This study used RNA-Seq data to investigate differential gene expression in the pituitary of South African Boer and Village goats that were raised under different production systems. The study hypothesised based on the growth rates, meat yield and meat quality analysis from previous studies (Ncube et al., 2016; Ncube et al., 2022) that the South African Boer goats raised intensively will be associated with upregulation of genes that promote higher growth rate and meat yield while downregulation of growth promoting genes would be expected in the village goats raised extensively.

## Materials and methods

### Animals and management

**FIGURE 1:**
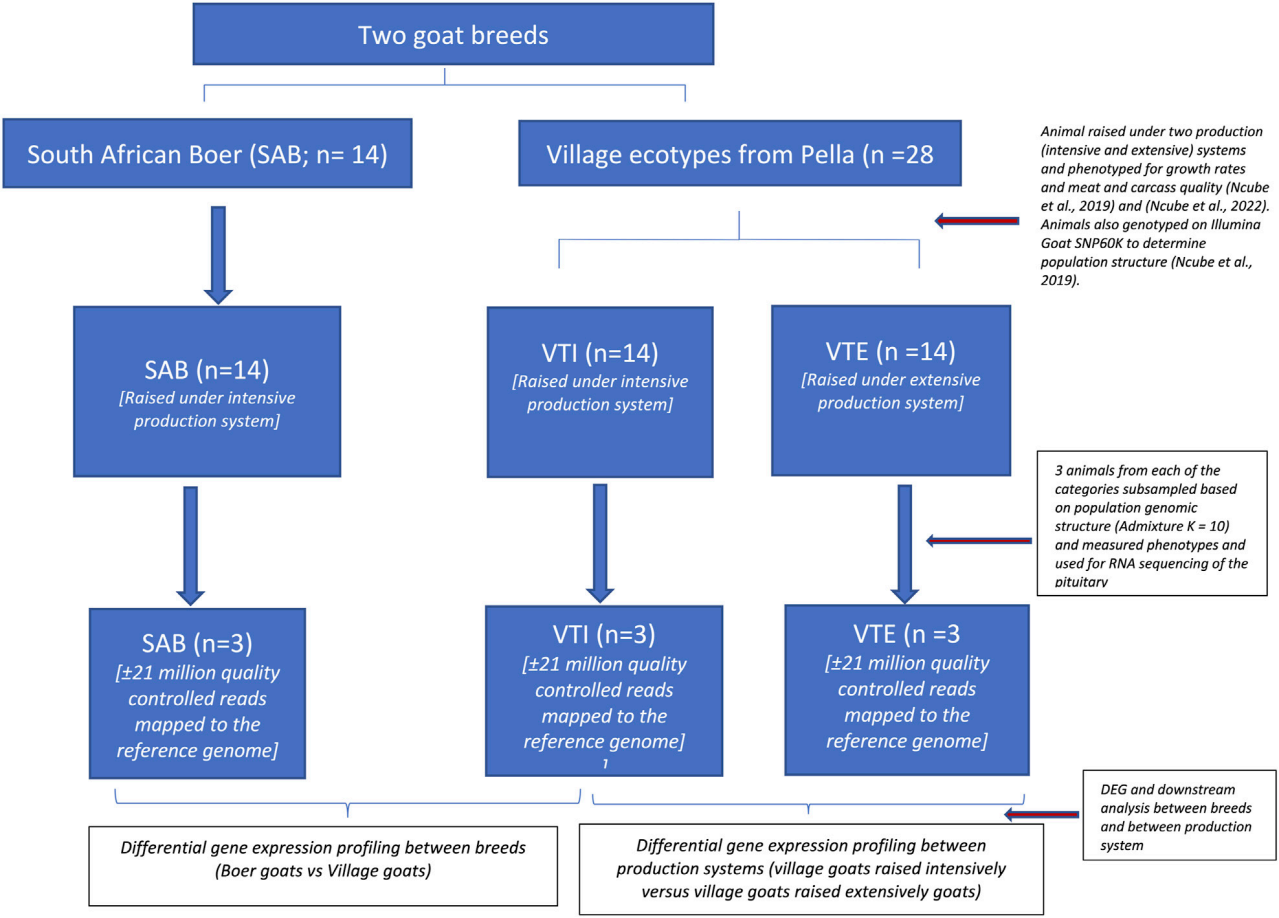
Experimental framework.

Two separate differential gene expression experiments were designed to study the impact of 1) breed differences within a production system and 2) production systems within a breed in the South African goat populations. Goats were sampled from the South African Boer (SAB, n = 14) and non-descript village populations that were procured from Pella village and divided into two subgroups one of which was raised under the intensive (VTI) and the second group raised under the extensive production system (VTE). The complete experimental layout is represented in Figure 1.

#### Experiment 1: Between breeds and within production system analysis

The experiment to investigate breed differences within a production system used SAB goats and non-descript village goats that were both raised under the same intensive production system. The SAB goats (*n* = 14) that were purchased from Indigenous Veld Goat Society members and the VTI goats (*n* = 14) procured from Pella that were both raised intensively, at the Agricultural Research Council, Animal Production (ARC-AP) under controlled feed and management conditions. The animals were kept in a grazing camp on management diet of game pellets (110 g/kg, 25–70 g/kg crude fat, 110–200 g/kg crude fibre, 6–10 g/kg calcium, 2.5 g/kg phosphorus and 3.68% non-protein nitrogen) provided at 3% of live weight /animal /day. Lucerne hay and clean water were available *ad libitum*.

#### Experiment 2: Within breed across production system analysis

South African village goats (*n* = 28) were sampled from 14 farms in Pella village, North West Province of South Africa. These 28 animals were split into two groups of 1) the extensive system (VTE) (*n* = 14) and 2) the intensive system (VTI) (*n* = 14), The VTE goats were purchased and raised in their respective village farms under extensive communal farming conditions in Pella. Goats kept at Pella village farms were penned at night and left to browse during the day with no supplementation provided in an arid environment characterised by low rainfall, little and poor quality pasture (Holmgren et al., 2006; Mdladla et al., 2017).

The growth trial to investigate the growth profiles and meat and carcass quality of the goats used in these two experiments was performed from October 2016 to March 2017 and reported by Ncube et al. (2019) and Ncube et al. (2022). In summary, the weaning (12 weeks) weights ranged from 9.00 to 16.40 kg for intensively raised and 7–13 kg for extensively raised VTI goats. The VTI had higher (*P< 0.005*) live weights at 36 weeks (31.00–33.00 kg) as compared to the population that was raised extensively at Pella village (22.50–28.00 kg) (Ncube et al., 2019). The carcass yield for the VTE were also lower (*P< 0.05*) than that of the VTI and SAB goats (Table 1; Ncube et al., 2022).

**TABLE 1:** Body weights of samples used at three different growth stages (12, 24, 36 weeks) for SAB, VTI and VTE.

Sample ID	Breed	Production system	12 W (kg)	24 W (kg)	36 W (kg)
16–191	Boer	Intensive	20	28.5	41
16–224	Boer	Intensive	15	25.5	39.5
16–225	Boer	Intensive	15	28	42
Mean ± SD (SAB)	Boer	Intensive	16.67 ± 2.89^a*^	27.33 ± 1.60^b*^	40.83 ± 1.26^c*^
PAPI01	Village	Intensive	9	22.5	32
PAPI03	Village	Intensive	10.5	19.5	31
PAPI04	Village	Intensive	16.4	24	33
Mean ± SD (VTI)	Village	Intensive	11.97 ± 3.91^a**^	22.00 ± 2.29^b**^	32.00 ± 1.00^c**^
P01	Village	Extensive	7	16	22.5
P03	Village	Extensive	13	20.5	28
P04	Village	Extensive	13	15	27.5
Mean ± SD VTE)	Village	Extensive	11.00 ± 3.46^a**^	17.17 ± 2.93^b***^	26.00 ± 3.04^c***^
Between breed analysis (Mean ± SD)			13.21 ± 3.03	22.12 ± 5.07	32.94 ± 7.51

^abc^Mean body weights with different superscript were significantly different at different age groups at (*p* < 0.05).

***Mean body weights with different superscript were significantly different amongst SAB, VTI, and VTE, at (*p* < 0.05).

#### Sample collection

Animals were humanely euthanized at 36 weeks and tissue harvesting was completed within 20 min post euthanasia to preserve RNA quality. Tissue samples were collected from the pituitary gland, which is located at the base of the goat brain. The samples were immediately stored in tubes containing the Qiagen RNA stabilization reagent, RNAlater (www.qiagen.com), to preserve the RNA and prevent degradation. The tissue samples were subsequently kept in a -80°C freezer.

#### RNA isolation and quantification

RNA samples were selected for sequencing based on the population genomic structure, which were derived from ADMIXTURE analyses using genotypic data from the OvineSNP50K Beadchip (Illumina Inc., San Diego, CA) (Ncube et al., 2019). From the ADMIXTURE analysis, three animals that clustered within the defined population cluster as observed at K = 10 (Ncube et al., 2019) were selected for each of the South African Boer (SAB), extensively raised village populations (VTE) and, intensively raised village populations (VTI). RNA isolation from 150 mg of the pituitary gland was performed using the Qiagen RNeasy and Qiagen RNA Universal Midi kit for purification of total RNA following the manufacturer’s instructions with slight modifications. The modifications included using 150 mg of tissue sample and homogenization was performed on the 2010 Geno/Grinder^®^ - Automated Tissue Homogenizer and Cell Lyser. RNA was eluted using 100 µl of RNA-free water. The quality of the RNA template was investigated electrophoretically on 1.8% agarose gel with ethidium bromide of 0.5 μg/ml at 80 V for 30min. The gel was examined under UV light for RNA degradation in a BIORAD Imaging System (BIORAD GelDoc XR) (www.bio-rad.com). The samples were submitted to the Agricultural Research Council, Biotechnology Platform (ARC-BTP) Core Facility for sequencing.

#### RNA sequencing

The cDNA libraries were prepared using an Illumina TruSeq Stranded Total RNA Ribo-Zero H/M/R Gold library prep kit (Illumina Inc., San Diego, CA) preparation. TruSeq universal adapters were ligated to the cDNA fragments, and PCR was performed to produce the final sequencing libraries. RNA was fragmented and randomly primed for reverse transcription to generate double-stranded cDNA fragments. Gel electrophoresis was used to assess the quality of the starting material. The cDNA was colligated, nebulized and then fragmented after which, adapters were ligated to both ends of the fragmented nucleic acid. The fragments were hybridized to a flow cell, which extended a hybridized template or performed bridge amplification. Sequencing was then performed using Illumina HiSeq 2,500. All cDNA samples for each animal were sequenced and examined in triplicates.

### Data analysis

#### RNA sequence trimming and quality control

Quality Control (QC) was performed on raw sequencing reads using Trimmomatic v0.36 (Bolger et al., 2014). Sequence trimming included the removal of the sequencing adapters, short reads as well as reads containing over 10% unknown bases (N), resulting in clean reads longer than 30 bp.

#### Sequence read alignment, assembly and, quantification

Reads that remained after QC were aligned and mapped to the goat genome (*Capra_hircus.ARS1.94*) from ENSEMBL (https://www.ensembl.org/Capra_hircus/) using HISAT2 v2.1.0 (Pertea et al., 2016). Genome indexing was performed with a maximum number of multiple hits set to 20 and non-mismatches at splice sites. Mapped reads were then sorted and converted to BAM files using SAMtools v1.3.3 (Pertea et al., 2016).

Transcript assembly and quantification from RNA sequence reads was performed with StringTie (Pertea et al., 2016). The transcripts abundance were measured in Fragments per Kilobase of transcript per Million mapped reads (FPKM) giving normalised transcripts produced from each individual genes relative to gene lengths and thus allowed for transcripts from genes of different length to be compared.

### Differential gene expression

For analysis of differentially expressed genes between experimental groups, the DESeq2 R package (Bioconductor, Buffalo, USA) was used with R v3.5.2. Genes with an FDR ≤0.05 and a log2 fold change of ≥2 and a Benjamini and Hochberg’s adjusted *p*-value ≤ 0.05 using were considered to be differentially expressed genes between breeds (SAB vs. VTI) and production systems (VTE vs. VTI). A volcano plot for highly significant genes was performed. Certain genes of interest that were not significantly expressed based on DESeq2 analysis were further analysis using Ballgown analysis (Pertea et al., 2016) using the FPKM values.

### Functional annotations and pathway analysis of differentially expressed genes

To gain more insight into the biological processes and implications of the differentially expressed genes (DEGs), gene ontology (GO) annotation and gene enrichment analyses were performed. GO analysis of DEGs was performed using the DAVID v6.7 online server (http://david.abcc.ncifcrf.gov/ (Huang et al., 2009)). DEGs were further mapped into the KEGG pathway database using KEGG mapper within the Kyoto Encyclopaedia of Genes and Genomes databases (KEGG, http://www.genome.jp/kegg) (Kanehisa, 2003) to predict the significantly enriched pathways. The DEGs of the groups under study were classified into cellular component, biological process and, molecular functions.

## Results

### RNA sequencing and read mapping

A minimum of 84 800 000 reads/animal were generated, and approximately 88.50% of the trimmed and quality-controlled reads were mapped to the goat reference genome (*Capra_hircus.ARS1.94*).

### Differential gene expression

#### Differential gene expression between breeds raised under the intensive production system

**FIGURE 2:**
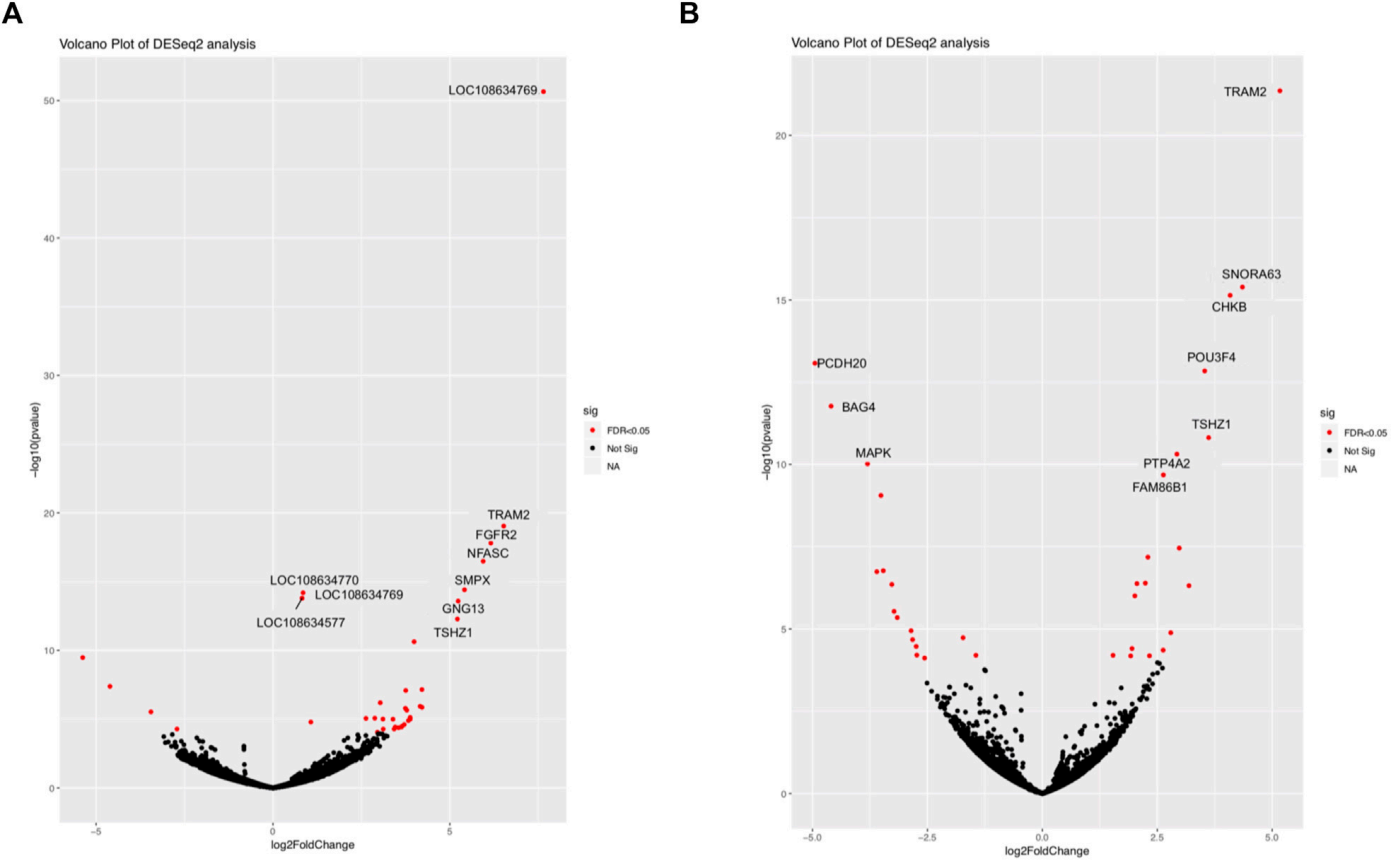
Volcano plot of differentially expressed genes between **(A)** SAB vs. VTI and **(B)** VTE vs. VTI. Highly expressed genes are shown in red.

The comparison between SAB and VTI goats revealed 51 674 expressed genes in 3 samples each of SAB and VTI goats, of which 45 005 were retained after filtering. Differentially expressed gene distribution is shown in a volcano plot (Figure 2A) in which the top 10 highly significant genes were labelled. Sixty-two of the expressed genes were differentially expressed from which 34 (54.8%) were up-regulated and 28 (45.1%) down-regulated. Highly differentially expressed genes included *TRAM2, FGFR2. NFASC, SMPX, TSHZI and NFASC* (Figure 2A; Supplementary Table S1.1).

#### Differential gene expression within the village goat breed and across production systems

Differentially expressed genes were considered as those with an FDR ≤0.05, a log2 fold change of ≥2, and a *p*-value ≤ 0.05. For within-breed DE, a total of 51,674 genes were expressed in the extensively (VTE) and intensively (VTI) raised village goats, and 40,964 were retained after filtering. Of the 40,964 expressed genes, 104 genes were differentially expressed, out of which 46 were up-regulated, and 58 were down-regulated. Highly differentially expressed genes included *TRAM2, SNORA63, CHKB, POU3F4, TSHZ1, PTP4A2* and *FAM86B1* (Figure 2B and Supplementary Table S1.2).

### Differential expression of genes of interest associated with growth

**FIGURE 3:**
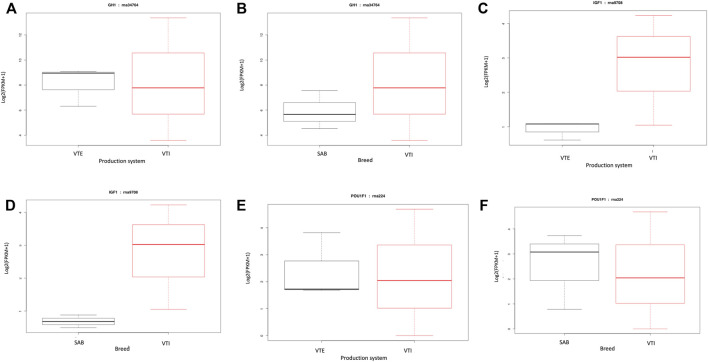
FPKM distributions of **(A)** *GH1* gene for VTE vs. VTI goats; **(B)** *GH1* gene for SAB vs. VTI; **(C)** *IGF1* gene for VTE vs. VTI; **(D)** *IGF1* gene for SAB vs. VTI; **(E)**) *POU1F1* gene for VTE vs. VTI; and **(F)** *POU1F1* gene for SAB vs. VTI goats displayed as box plots.

Certain genes are known to play a significant role in the growth of animals, and this study hypothesized that these genes would be among the most significantly expressed genes. Some of these key genes included *GH1*, *IGF1* and *POU1F1* that work as a network to influence the growth of animals. However, these genes were expressed at lower fold change (≤2) ranging from 0.90 to 0.95 with a *p*-value from 0.61 to 0.83. The *Growth hormone 1* gene, also known as the pituitary growth hormone, is a significant participant in control of several complex physiological processes, including growth and metabolism. This gene was moderately upregulated in the VTI goats relative to the VTE (Figure 3A) and SAB (Figure 3B). The *IGF1* gene that is a mediator of the effects of GH1, stimulates systemic body growth and has growth-promoting effects. The IGF1 gene was, like GH1, moderately upregulated in the VTI goat as compared to the extensively raised VTE (Figure 3C) and the SAB (Figure 3D). The pituitary specific transcription factor 1 (POU1F1) gene activates growth hormone and it is responsible for pituitary development and hormone expression in mammals. This gene was moderately upregulated in the pituitary tissue for the VTI goats while moderately expressed in the VTE animals (Figure 3E) and SAB goats (Figure 3F).

### Functional annotations of differentially expressed genes

**FIGURE 4:**
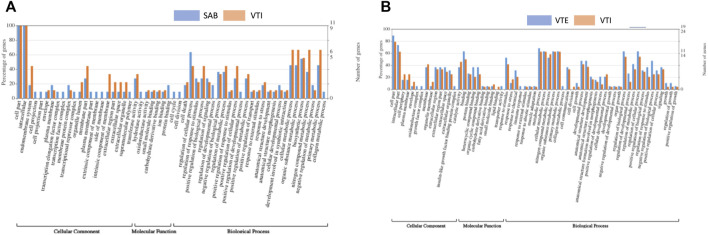
Gene ontology (GO) of differentially expressed genes **(A)** SAB and VTI goats and [**(B)** VTE and VTI]. The right *y*-axis indicates the number of genes in a category. The left *y*-axis indicates the percentage of a specific category of genes in that main category. One gene could be annotated into more than one GO term.

To better understand gene networks, gene ontology (GO) annotation was performed using Fisher’s exact test and filtering using multiple correlation controls for false discovery rate (FDR) ≤ 0.05. Gene ontology (GO) terms are typically described by three functional groups, cellular component (CC), biological process (BP) and molecular function (MF). The GO terms were associated with differentially expressed genes in both SAB vs. VTI and VTI x VTE as shown in Figures 4A,B. Functional annotation analysis between SAB and VTI goats resulted in cellular components, biological processes, and molecular functions (Figure 4A). There were no significantly enriched GO terms observed within this analysis. However, of all the DEGs that were identified only in the SAB population, those that play a role in the cellular component such as the cell projection, cell projection part, transcription elongation factor complex, transcription factor complex, plasma membrane part, extrinsic component of membrane, side of membrane and supramolecular polymer were also observed. Cell cycle and cell division molecular functions were also observed only in the SAB population. The GO analysis revealed DEGs that play a role in the biological processes such as regulation of developmental process, positive regulation of biological process, anatomical structure development, anatomical structure morphogenesis and cellular developmental process involved in symbiotic interactions in both the SAB and VTI populations except for positive regulation of biological process which was observed only in the SAB goats.

In the analysis that compared VTE versus VTI goat ecotypes, significantly annotated gene ontology (GO) terms were within the three major functional groups, cellular component (CC), biological process (BP) and molecular function (MF) (Figure 4B). Some of the differentially expressed genes (DEGs) that play a role in the cellular component such as insulin like growth factor binding protein complex, oxidoreductase complex and growth factor complex were observed only in the extensively raised populations. Lipid binding molecular function was observed only in the VTE populations. GO analysis revealed DEGs that play a role in the biological processes such as muscle adaptation, cell growth, anatomical structure development, anatomical structure morphogenesis involved in morphogenesis, developmental process and growth as well as organ growth in both the VTE and VTI goats. The five most significantly enriched GOs were observed in the biological processes category (Figure 4A), negative regulation of biological process and regulation of response to stimulus, under the molecular functions category were the heterocyclic compound binding, and organic compound cyclic binding and binding was under the cellular components category.

### Pathway analysis

For an understanding of biological pathways that are involved between breeds and different production systems, the KEGG pathway enrichment analysis was performed using the DAVID 6.7 Functional Annotation Tool (Huang et al., 2009) (http://david.abcc.ncifcrf.gov/) (Table 2, 3).

**TABLE 2:** Pathway analysis of differential gene expression within breeds raised under different production systems (extensively raised *vs*. intensively raised) village goat populations.

Pathway	Gene symbol	Gene name	Fold enrichment	Benjamini	*p* Value
Metabolic	ACADVL; CHKB; DPM3: GNPDA1; RRM2B	Acyl-CoA dehydrogenase, very long chain; choline kinase beta; dolichyl-phosphate mannosyltransferase subunit 3; glucosamine-6-phosphate deaminase 1; ribonucleotide reductase regulatory TP53 inducible subunit M2B	2.18	0.81	0.15
RNA degradation	EXOSC3; PAPD7	Exosome component 3; poly(A) RNA polymerase D7, non-canonical	14.19	0.87	0.12
p53 signalling	IGFBP3; RRM2B	Insulin like growth factor binding protein 3; ribonucleotide reductase regulatory TP53 inducible subunit M2B	15.19	0.98	0.12

**TABLE 3:** Pathway analysis of differential gene expression between breeds raised under the same production system (Boer vs. village) goat populations.

Pathway	Gene symbol	Gene name	Fold enrichment	Benjamini	*p* Value
Metabolic	DPM3; MAN1C1; NDUFB7	Dolichyl-phosphate mannosyltransferase subunit 3; mannosidase alpha class 1C member 1; NADH:ubiquinone oxidoreductase subunit B7	2.62	2.93	0.26
N-Glycan biosynthesis	DPM3; MAN1C1	dolichyl-phosphate mannosyltransferase subunit 3; mannosidase alpha class 1C member 1	44.02	0.77	0.04
PI3K-Akt signalling	GNG13; PRKAA2	G protein subunit gamma 13; protein kinase AMP-activated catalytic subunit alpha 2	6.22	0.97	0.25
Non-alcoholic fatty liver	NDUFB7, DPM3, MAN1C1	NADH:ubiquinone oxidoreductase subunit B7; dolichyl-phosphate mannosyltransferase polypeptide 3; mannosidase alpha class 1C member 1	13.48	0.90	0.12

**FIGURE 5:**
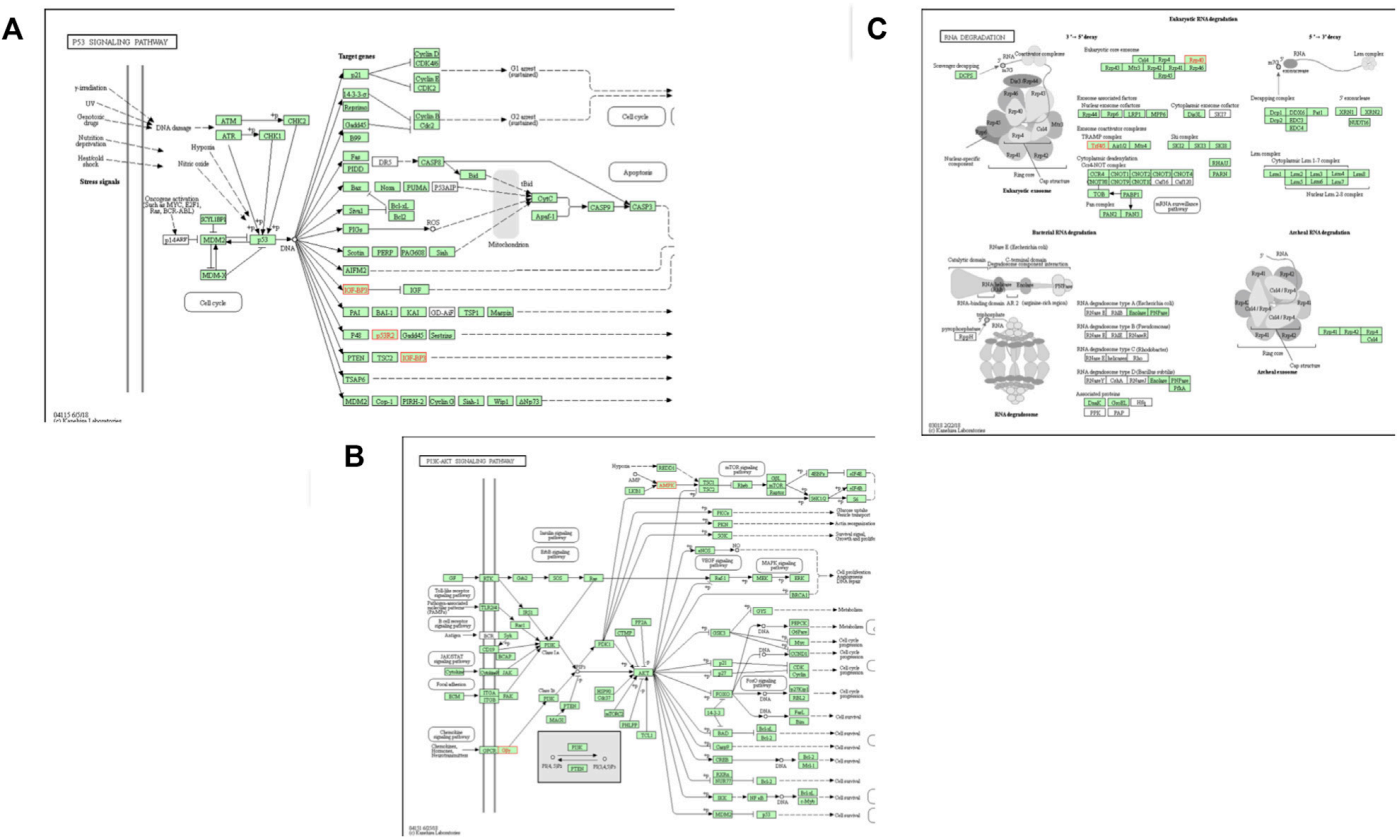
Top three enriched pathways of **(A)** p53 Signalling pathway **(B)** RNA degradation pathway and **(C)** PI3K-Akt signalling pathway. Genes involved are highlighted in red.

In the SAB vs. VTI goat pathway analysis, the four enriched pathways were metabolic, N-glycan biosynthesis, PI3K-Akt signalling and Non-alcoholic fatty liver pathways. Three genes (*DPM3, MAN1C1,* and *NDUFB7*) were enriched in both the metabolic and Non-alcoholic fatty liver pathways. Two genes (*DPM3* and *MAN1C1*) were enriched in the N-glycan biosynthesis pathway and two other genes (*GNG13* and *PRKAA2*) were enriched in the PI3K-Akt signalling pathway (Figure 5A). The top three enriched biological pathways reported in the comparison of VTE vs. VTI village goats were metabolic, p53, and RNA degradation pathways. Five genes (*ACADVL, CHKB, DPM3, GNPDA1,* and *RRM2B*) were enriched in the metabolic pathway. Two genes (*IGFBP3* and *RRM2B*) were enriched in the p53 pathway (Figure 5B) and two other genes (*EXOSC3* and *PAPD7*) were enriched in the RNA degradation pathway (Figure 5C).

## Discussion

Growth is an essential quantitative trait that influences mature bodyweights and the overall productivity of the goat production system (Supakorn, 2009). In a country where a large number of goats are raised in impoverished, marginalized communal production communities, there exist goat populations that are distinguished for their adaptability to local conditions and low input production systems (Dzomba et al., 2018). South Africa has established and developed meat breeds of which the Boer goat is one. This breed is well known for its high meat yield and growth performance and is mainly raised under large scale intensive commercial systems (Pieters, 2007). Village goats, on the other hand, are uncharacterized populations that are raised extensively in communal areas commonly known for poor growth performance. This study hypothesized that the production system and breed differences are some of the critical factors that influence growth and carcass quality due to underlying genetic mechanisms. The study analysed between breed differences by comparing South African Boer goat raised intensively (SAB) versus village goats raised intensively (VTI), whilst between production system differences were analysed by comparing village goats raised intensively (VTI) to those raised extensively (VTE). One shortfall of this study is the failure to compare Boer goats raised in the villages with Boer goats raised intensively. This analysis would have provided a complete picture of the effect of production system on the performance of a breed and should be pursued.

Few DEGs were observed between SAB and VTI goats, which were both raised under intensive production system, and DEGs that play a role in the growth and development of goats were identified (*FGFR2, NFASC, SMPX* and *LOC108634577*). The *fibroblast growth factor receptor 2 gene* (*FGFR2*) play a vital role in bone growth during embryonic development. It encodes a protein that is a member of the fibroblast growth factor receptor family which signals some immature cells in the developing embryo to become bone cells and form organs such as the head, hands, feet, and other organs. This gene was up-regulated in the SAB goats that are a higher performing breed with an average 100-days weaning weight of 27 kg (Snyman, 2014) as compared to VTI with an average weaning weight of 13 kg (Ncube et al., 2019). The *small muscular protein* (*SMPX*) gene, on the other hand, is involved in the regulatory network through which muscle cells coordinate their structural and functional states during growth, adaptation and repair. It was observed to be expressed in skeletal muscle where it plays a role in protecting sarcolemmal plasma membrane from mechanical stress (Abdelfatah et al., 2013). *SMPX* gene can also be found in the myotomal compartment of somites and in developing limb, head and neck muscles in adult mice where it was highly expressed in heart chambers and skeletal muscle (Palmer et al., 2001). The high expression of *SMPX* would then be associated to high muscle development and therefore expected in the SAB which, is associated with high meat yield. Both *FGFR2* and *SMPX* are involved in prenatal growth processes and their high expression in SAB that is associated with high postnatal growth rate and meat yield is in agreement with reports by Greenwood and Café (2007) that growth rates and retail meat yield in livestock is directedly influenced by growth and development during pregnancy or from birth to weaning.

A high number of genes was differentially expressed between VTI and VTE goats. DEGs (*SNORA63, CHKB, POU3F4, MAPK* and *BAG4*) that play a crucial role in growth and development were identified. Small Nucleolar RNA, H/ACA Box 63tbox63 (*SNORA63*) is part of the H/ACA class of snoRNAs and is involved in the processing of eukaryotic pre-rRNA where the E3 is encoded in introns in the gene for protein synthesis initiation factor 4AII. The *SNORA63* gene has been identified in various developmental stages and in different organs in vertebrates (Kehr et al., 2014), and results from this study suggests that this gene is only involved in the development of South African village goats. Goshu et al. (2019) indicated a significant association of *Chlorine kinase beta* (*CHKB*) with growth traits such as chest girth and bodyweight of Datong yaks suggesting that this gene could be a novel marker for growth traits and can be used in genomics assisted selection in animal breeding.

The *POU domain, class 3, transcription factor 4* (*POU3F4*) is involved in the patterning of the neural tube and both the paraventricular and supraoptic nuclei of the hypothalamus in the developing embryo. The hypothalamus is located near the pituitary gland and plays a role in the endocrine system by releasing hormones such as the growth hormone-releasing hormone (GHRH) that stimulates the pituitary gland to release the growth hormone, or the growth hormone inhibiting hormone (GHIH) which has the opposite effect. The downregulation of this gene will then inhibit the growth hormone from being released; therefore, negatively affecting the growth of animals while up-regulation will aid in the growth of an animal. High expression of this gene in the VTE goats leads to growth hormone inhibition, leading these goats to be slow-growing with low mature bodyweights. *Mitogen activated protein kinase* (*MAPK*), on the other hand, directs cellular responses to a variety of stimuli such as proinflammatory cytokinesis, mitogenesis, osmotic stress, etc. And also regulates cell functions such as proliferation, gene expression, differentiation, cell survival and apoptosis. MAPK is highly expressed and up-regulated in the VTE animals indicating that village goat populations are under a greater amount of stress inherent in most communal farming systems set ups. BCL2 associated athanogene 4 (*BAG4*) is one of the growth-related genes that interacts with BAG1, which is an anti-apoptotic protein. BAG4 is upregulated in the VTI goats and has been identified as a candidate gene for residual feed intake in Holstein cows (Hardie et al., 2017). The present study suggests that the BAG4 gene has potential as a candidate gene for growth in South African populations which is consistent with the Holstein study [29].

Some of the DEGs though not significantly expressed, are known for their significant impact on growth and development including, *GH1*, *IGF1* and *POU1F1*. As a major participant in control of several physiologic processes including growth and metabolism, the *GH1* gene also known as the pituitary growth hormone is a member of a family of hormones that are secreted in the anterior pituitary gland including growth hormone which the primary function is to stimulate growth in all tissues of the body including bones. The VTI goats showed a growth performance improvement under improved management systems which explains why this gene was more expressed in intensively raised as compared to extensively raised populations. The *GH1* gene was reported to be highly polymorphic within village populations and was suggested as a candidate gene for genomics assisted breed selection and improvement programs (Ncube et al., 2016).

The effects of growth hormone are primarily mediated by the *IGF1* gene, which stimulates body growth and has growth-promoting effects in almost every cell of the body, such as skeletal muscle, bone, etc. Growth hormone is produced in the pituitary gland then released into the bloodstream where it then stimulates the liver to produce *IGF1*. Expression in the VTE was extremely low compared to VTI suggesting that its function of mediating growth hormone effects that promote growth in animals is inhibited, therefore leading to poor growth performance. Another reason for low expression may be that, though it is stimulated by the growth hormone from the pituitary gland, it is primarily produced in the liver. On the other hand, relatively high expression in the intensively raised populations further supports the hypothesis that improved management systems lead to improved growth performance.

Between breed differences were investigated by comparing the SAB to VTI goats raised under the same production system. The study observed that there were more genes expressed in the SAB goats than in the village goat population. The Boer is a well-developed breed that was developed from the indigenous goat populations as a meat breed (Mdladla et al., 2017). This breed was reported as a fast-growing and high performing breed (Dzomba et al., 2018) and the high expression of genes in the Boer compared to village populations may be due to the fact that village populations have not been selected for high growth and meat performance. Overall, there were more up-regulated genes reported in the VTE goats, which might be due to the extreme and diverse environmental conditions experienced by animals in the village farming community (Masika and Mafu, 2004; Webb and Mamabolo, 2004).

The gene ontology (GO) analysis in this study revealed DEGs that play a role in cell components such as growth factor and insulin growth factor-binding protein complex in the extensive production system. Biological processes affected included processes such as cell growth, developmental and organ growth, negative regulation of developmental process, growth, regulation of growth, anatomical structure development, developmental process, anatomical structure morphogenesis and anatomical structure formation involved in morphogenesis which were observed in both the extensive and intensive production systems. The p53 signaling pathway observed within-breeds raised under different production systems is induced by several stress signals, including RNA damage and oxidative stress which are expected in extensively raised populations. Two genes, *insulin like growth factor-binding protein 3* (I*GFBP3*) and *ribonucleic reductase regulatory TP53 inducible subunit M2B* (*RRM2B*) were enriched in the p53 signaling pathway, and these were upregulated in the VTE goats. The *IGFBP3* gene plays an essential role in the regulation of postnatal somatic growth and as well as the stimulation of anabolic processes (Ramesha et al., 2015). The *IGFBP3* is one of the proteins that bind to insulin growth factors (IGF) which play a vital role in the regulation of cell proliferation and apoptosis (Ramesha et al., 2015). Up-regulation of *IGFBP3* would lead to cell apoptosis, therefore, causing reduced growth performance in animals that are raised in harsh conditions.

## Conclusion

In summary, this study was able to employ RNA-Seq technology in the identification of differentially expressed genes in the pituitary gland of the South African SAB and Village goat populations raised under intensive and extensive production systems. Genes associated with embryonic development were differentially upregulated in SAB versus VTI suggestive of role of prenatal growth and development in postnatal growth and meat yield and quality. Growth-related genes such as the *CHKB, POU3F4, BAG4* and *IGF1* were highly expressed in the VTI versus VTE demonstrating the positive impact of production systems on the realisation of genetic potential in village goats. More DEGs were observed in the VTI vs. VTE comparison suggestive of the broad range of genetic mechanisms required for survival and production of goats under the smallholder village production systems.

## Data Availability

The Bioproject for the sequence data generated and used in this study is publicly available at https://www.ncbi.nlm.nih.gov/bioproject/PRJNA861398 with the accessions numbers: SAMN29912371, SAMN29912372, SAMN29912373, SAMN29912374, SAMN29912375, SAMN29912376, SAMN29912377, SAMN29912378 and SAMN29912379.
