# Supplementary material for: Differential gene expression and identification of growth-related genes in the pituitary gland of South African goats
Source: Front Genet. 2022 Aug 22;13:811193. doi: 10.3389/fgene.2022.811193 (PMC9442344; doi:10.3389/fgene.2022.811193)
Supplement: Supplementary file 1 [file Table1.DOCX]

**Supplementary Table S1.4**: Differential gene expression of SAB goat populations in the VTI vs SAB comparison

| Gene Name | Gene Description | Log2 FC | FDR | *P*_value |
| --- | --- | --- | --- | --- |
|  |  |  |  |  |
| **SAB Up-Regulated** |  |  |  |  |
| MAPRE2 | Microtubule associated protein RP/EB family member 2 | 11.17780 | 0.00008 | 3.17E-08 |
| NDUFB7 | NADH:ubiquinone oxidoreductase subunit B7 | 4.28949 | 0.00303 | 2.29E-06 |
| TRAM2 | Translocation associated membrane protein 2 | 13.67044 | 1.01E-11 | 8.97E-16 |
| ELP2 | Elongator acetyltransferase complex subunit 2 | 18.16198 | 0.00021 | 8.85E-08 |
| FMNL2 | Formin like 2 | 9.15837 | 0.00146 | 9.08E-07 |
| MLIP | Muscular LMNA interacting protein | 10.20452 | 0.01183 | 1.10E-05 |
| GNG13 | G protein subunit gamma 13 | 12.56977 | 1.01E-11 | 7.45E-16 |
| NRCAM | Neuronal cell adhesion molecule | 6.65436 | 0.02848 | 0.00003 |
| PPIP5K2 | Diphosphoinositol pentakisphosphate kinase 2 | 10.42396 | 0.00004 | 1.49E-08 |
| NCOR2 | nuclear receptor corepressor 2 | 8.50446 | 0.04831 | 6.66E-05 |
| NKX2-1 | NK2 homeobox 1 | 9.78257 | 0.00022 | 1.01E-07 |
| PPIAP11 | Peptidylprolyl isomerase A pseudogene 11 | 5.31748 | 0.00094 | 5.20E-07 |
| NEDD4 | NEDD4 E3 ubiquitin protein ligase | 6.15567 | 0.01827 | 1.91E-05 |
| TMCC1 | Transmembrane and coiled-coil domain family 1 | 3.34148 | 0.02177 | 0.00002 |
| TFDP2 | Transcription factor Dp-2 | 8.94258 | 0.03199 | 0.00004 |
| USP32 | Ubiquitin specific peptidase 32 | 9.20405 | 0.007266 | 6.46E-06 |
| BMPR1B | Bone morphogenetic protein receptor type 1B | 9.71264 | 0.00006 | 2.16E-08 |
|  |  |  |  |  |
| **SAB Down-Regulated** |  |  |  |  |
| CD38 | Cluster of differentiation | -4.15993 | 0.01467 | 1.43E-05 |
| MYZAP | Myocardial zonula adherens protein | -11.20905 | 0.00003 | 1.08E-08 |
| MAN1C1 | Mannosidase alpha class 1C member 1 | -10.07764 | 0.04608 | 6.04E-05 |
| PRKAA2 | Protein kinase AMP-activated catalytic subunit alpha 2 | -12.62486 | 0.00254 | 1.75E-06 |
| TRDN | Triadin | -5.28741 | 0.00223 | 1.49E-06 |
