# Supplementary material for: Differential gene expression and identification of growth-related genes in the pituitary gland of South African goats
Source: Front Genet. 2022 Aug 22;13:811193. doi: 10.3389/fgene.2022.811193 (PMC9442344; doi:10.3389/fgene.2022.811193)
Supplement: Supplementary file 2 [file Table2.DOCX]

APPENDIX G: DIFFERENTIAL GENE EXPRESSION OF EXTENSIVELY RAISED VILLAGE GOAT POPULATIONS

| **Gene Name** | **Gene Description** | | | **Log2 FC** | | **FDR** | ***P*_value** | |
| --- | --- | --- | --- | --- | --- | --- | --- | --- |
|  |  | | |  | |  |  | |
| **Extensive System Up-Regulated** |  | | |  | |  |  | |
| MYDGF | Myeloid derived growth factor | | | 6.04970 | | 0.00858 | 0.00001 | |
| GNPDA1 | Glucosamine-6-phosphate deaminase 1 | | | 4.55493 | | 0.01862 | 0.00003 | |
| KDM5A | Lysine demethylase 5A | | | 10.16079 | | 0.04019 | 0.00009 | |
| DLG2 | Discs large MAGUK scaffold protein 2 | | | 11.32892 | | 6.13E-08 | 8.98E-12 | |
| ZFAND5 | Zinc finger AN1 type containing 5 | | | 11.31772 | | 3.44E-06 | 1.18E-09 | |
| TRAM2 | Translocation associated membrane protein 2 | | | 14.00741 | | 1.29E-16 | 2.63E-12 | |
| RRM2B | Ribonucleotide reductase regulatory TP53 inducible subunit M2B | | | 7.07406 | | 0.00055 | 4.36E-07 | |
| CELF2 | CUGBP Elav-like family member 2 | | | 5.73902 | | 0.00322 | 4.02E-06 | |
| LOC102173699 | 60S ribosomal protein L6 pseudogene | | | 3.94201 | | 0.04164 | 0.00010 | |
| PTP4A2 | Protein tyrosine phosphatase 4A2 | | | 12.16172 | | 9.91E-08 | 2.18E-11 | |
| EXOSC3 | Exome component 3 | | | 7.58079 | | 0.03296 | 0.00007 | |
| TPM1 | Tropomyosin 1 | | | 8.66019 | | 0.00197 | 2.26E-06 | |
| IGFBP3 | Insulin like growth factor binding protein 3 | | | 9.29734 | | 0.03296 | 0.00007 | |
| FMNL2 | Formin like 2 | | | 7.98313 | | 0.01311 | 0.00002 | |
| FAM86B1 | Family with sequence similarity 86-member B1 | | | 12.70133 | | 0.00015 | 1.05E-07 | |
| DST | Dystonin | | | 10.79967 | | 0.00003 | 1.34E-08 | |
| FAM85A | Family with sequence similarity 85-member A | | | 9.95793 | | 0.00315 | 3.84E-06 | |
| WNK1 | WNK lysine deficient protein kinase 1 | | | 3.28435 | | 0.04391 | 0.00011 | |
| PGRMC2 | Progesterone receptor membrane component 2 | | | 8.60815 | | 0.00195 | 2.14E-06 | |
| TRPC3 | Transient receptor potential cation channel subfamily member 3 | | | 8.28544 | | 0.00197 | 2.26E-06 | |
| MESTP2 | Mesoderm specific transcript pseudogene 2 | | | 4.69934 | | 0.01221 | 0.00002 | |
| ARFIP1 | ADP ribosylation factor interacting protein 1 | | | 9.29275 | | 0.01544 | 0.00002 | |
| NKX2-1 | NK2 homeobox 1 | | | 8.86752 | | 0.00055 | 4.39E-07 | |
| SNORA63 | small nucleolar RNA, H/ACA box 63 | | | 8.95258 | | 6.45E-07 | 1.73E-10 | |
| ENO1P4 | enolase 1 pseudogene 4 | | | 9.24184 | | 9.71E-07 | 2.84E-10 | |
| RBM22P2 | RNA binding motif protein 22 pseudogene 2 | | | 9.33545 | | 0.00067 | 5.53E-07 | |
| RABGAP1 | RAB GTPase activating protein 1 | | | 4.03513 | | 0.00664 | 8.91E-06 | |
| CAPRIN2 | Caprin family member 2 | | | 7.60697 | | 0.03332 | 0.00007 | |
| CHKB | Chlorine kinase beta | | | 12.78504 | | 4.00E-15 | 4.09E-11 | |
| SLC13A4 | Solute carrier family 13 member 4 | | | 8.85552 | | 0.03143 | 0.00006 | |
| LOC108637268 | Translation machinery-associated protein 7 pseudogene | | | 8.14000 | | 0.00324 | 4.12E-06 | |
| GIGYF1 | GRB10 interacting GYF protein 1 | | | 3.33623 | | 0.04571 | 0.00012 | |
| TFDP2 | Transcription factor Dp-2 | | | 8.84442 | | 0.00189 | 2.03E-06 | |
|  |  | | |  | |  |  | |
| **Extensive System Down-Regulated** |  | | |  | |  |  | |
| PAPD7 | Poly(A) RNA polymerase D7, non-canonical | | | -3.79685 | | 0.01354 | 0.00002 | |
| RTP4 | Receptor transporter protein 4 | | | -3.96664 | | 0.02571 | 0.00005 | |
| GREB1L | GREB1 like retonic acid receptor coactivator | | | -9.252529 | | 0.00004 | 2.01E-08 | |
| WBSCR16 | Williams-Beuren syndrome chromosome region 16 | | | -3.61153 | | 0.03296 | 0.00007 | |
| EDN1 | Endothelin 1 | | | -6.91612 | | 0.03929 | 0.00009 | |
| EFNA1 | Ephrin A1 | | | -4.45724 | | 0.02799 | 0.00005 | |
| ACADVL | acyl-CoA dehydrogenase very long chain | | | -5.87293 | | 0.00034 | 2.41E-07 | |
| SKP2 | S-phase kinase associated protein 2 | | | -4.84049 | | 0.04799 | 0.00012 | |
| MAPT | Microtubule associated tau | | | -6.06321 | | 0.00489 | 6.45E-06 | |
| ANKH | ANKH pyrophosphate transport regulator | | | -4.61531 | | 0.00154 | 1.43E-06 | |
| TP53INP1 | Tumour protein p53 inducible protein 1 | | | -4.32553 | | 0.03742 | 0.00009 | |
| AMN1 | Antagonist of mitotic exit network 1 homolog | | | -4.05730 | | 0.01863 | 0.00003 | |
| PDH20 | | Protocadherin 20 | -13.03849 | | 6.19E-13 | | 1.51E-17 |  |
|  |  | | |  | |  |  | |
| MAPK | Mitogen-activated protein kinase | | | -11.89218 | | 1.54E-06 | 4.89E-10 | |
| BAG4 | BCL2 associated athanogene 4 | | | -6.63597 | | 8.78E-08 | 1.50E-11 | |
